# Death of a dogma: eukaryotic mRNAs can code for more than one protein

**DOI:** 10.1093/nar/gkv1218

**Published:** 2015-11-17

**Authors:** Hélène Mouilleron, Vivian Delcourt, Xavier Roucou

**Affiliations:** 1 Department of biochemistry, Université de Sherbrooke, Sherbrooke, Quebec J1E 4K8, Canada; 2 PROTEO, Quebec Network for Research on Protein Function, Structure, and Engineering, Quebec, Canada; 3 Inserm U-1192, Laboratoire de Protéomique, Réponse Inflammatoire, Spectrométrie de Masse (PRISM), Université de Lille 1, Cité Scientifique, 59655 Villeneuve D'Ascq, France

## Abstract

mRNAs carry the genetic information that is translated by ribosomes. The traditional view of a mature eukaryotic mRNA is a molecule with three main regions, the 5′ UTR, the protein coding open reading frame (ORF) or coding sequence (CDS), and the 3′ UTR. This concept assumes that ribosomes translate one ORF only, generally the longest one, and produce one protein. As a result, in the early days of genomics and bioinformatics, one CDS was associated with each protein-coding gene. This fundamental concept of a single CDS is being challenged by increasing experimental evidence indicating that annotated proteins are not the only proteins translated from mRNAs. In particular, mass spectrometry (MS)-based proteomics and ribosome profiling have detected productive translation of alternative open reading frames. In several cases, the alternative and annotated proteins interact. Thus, the expression of two or more proteins translated from the same mRNA may offer a mechanism to ensure the co-expression of proteins which have functional interactions. Translational mechanisms already described in eukaryotic cells indicate that the cellular machinery is able to translate different CDSs from a single viral or cellular mRNA. In addition to summarizing data showing that the protein coding potential of eukaryotic mRNAs has been underestimated, this review aims to challenge the single translated CDS dogma.

## BACKGROUND—THE SINGLE FUNCTIONAL ORF PERSPECTIVE OF EUKARYOTIC mRNAS

**Figure 1.**
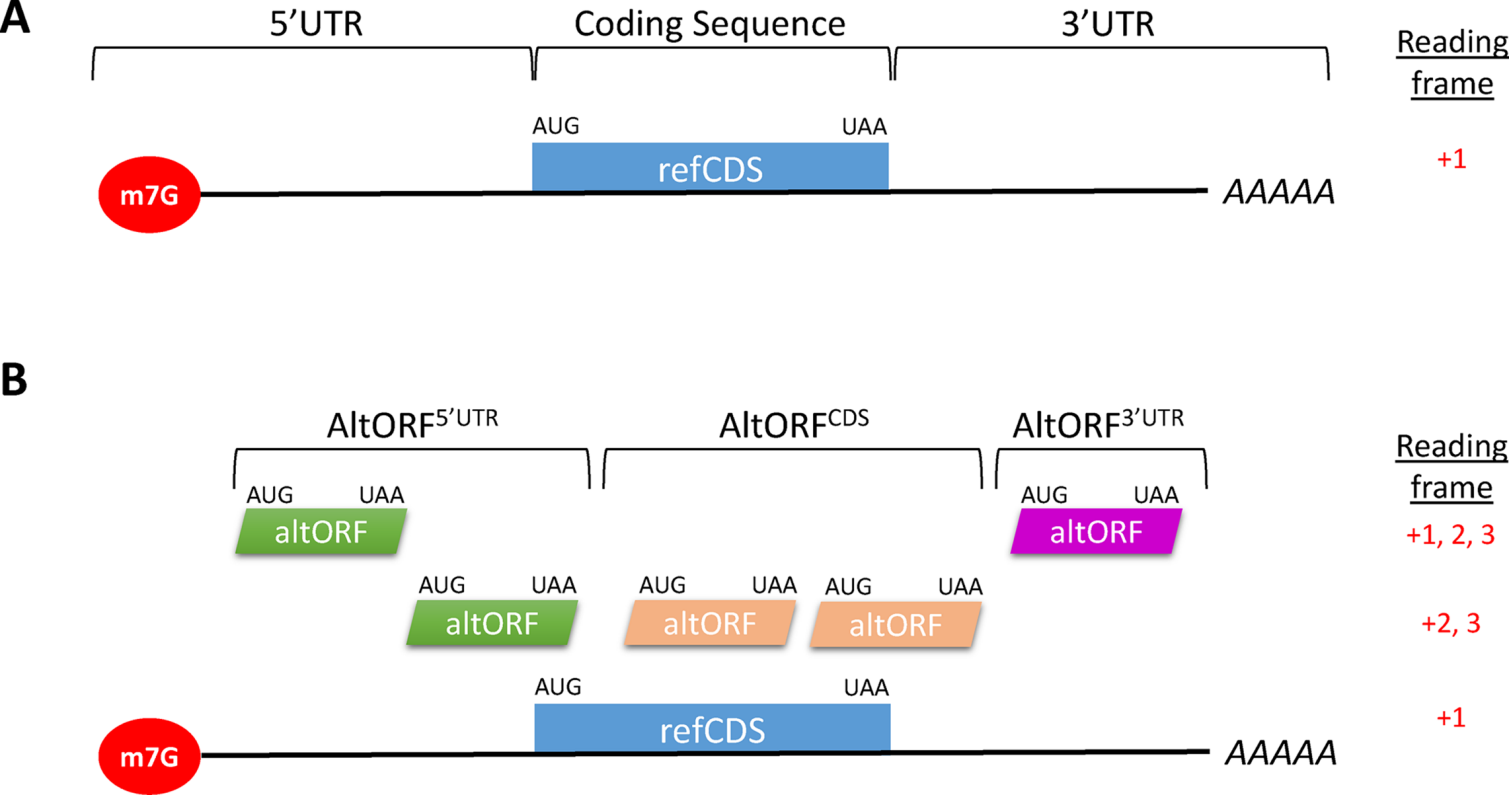
The typical tripartite structure of a eukaryotic mRNA with a single annotated or reference coding sequence or refCDS (**A**, single CDS dogma), or with possible alternative ORFs with an initiation codon located in the 5′ UTR, the CDS, or the 3′ UTR. (**B**). AltORFs^5′UTR^ may overlap refCDSs in a different reading frame. AltORFs^CDS^ may extend into 3′ UTRs. In general, refCDSs are longer than altORFs. Generally, all annotated mRNAs have a refCDS. An mRNA may have no, one or several altORFs. Abbreviations: AAAAA, polyA tail; altORF, alternative open reading frame; AUG, translation initiation codon; refCDS, annotated or reference protein coding sequence; m7G, 7-methyl-guanosine cap; UAA, stop codon (only one possible stop codon is shown for simplicity).

The general vision of a typical eukaryotic mature mRNA is a monocistronic molecule with a tripartite structure: a single translated ORF or CDS is flanked by 5′ and 3′ UTRs (Figure 1A). Although bicistronic mRNAs were detected in plants and some aspects of the translational mechanisms for these mRNAs have been elucidated (1), this review will mainly focus on animal mRNAs. The single CDS concept mainly stems from the canonical cap dependent scanning mechanism model for the selection of translation initiation sites in eukaryotic mRNAs. This model has provided a fundamental framework for the study of the regulation of translation initiation in eukaryotes and is supported by a substantial body of data (2–4). Briefly, a 43S preinitiation complex binds to the cap structure at the 5′ end and scans the 5′ UTR to arrest at a translation initiation site (TIS). The large 60S subunit then joins to form a fully functional 80S ribosome and polypeptide synthesis starts. In contrast to bacterial ribosomes which can bind to internal binding sites in polycistronic mRNAs, the cap-dependent scanning mechanism as currently visioned is not compatible with the translation of several CDSs in the same mRNA molecule. Consequently, it was concluded well before the pre-genomic era that each mature eukaryotic mRNA is monocistronic and is translated into a single polypeptide (5).

This dominant view of a single functional ORF intensified with the implementation of computational pipelines for automated annotation of genomes. Contemporary approaches to identify protein-coding genes in large eukaryotic genomes and transcriptomes generally use combinations of three methods: (i) statistical information, including codon usage; (ii) splice sites and sequence similarity to previously identified proteins and genes; and (iii) experimental evidence of transcript-derived sequences of cDNAs or expressed sequence tags (6–12). Fortunately, vast resources of cDNAs, ESTs, and protein sequences have been assembled in public databases and are invaluable for the analysis of large genomes (13). The refinement of the predictions of protein-coding genes using cDNAs and ESTs has clearly improved the accuracy of the annotation of CDSs within large genomes (14). Reviews and comparisons of several methods for the identification of protein-coding regions identification methods are available (15–21). In the absence of experimental data on gene expression or gene homology, several programs can be used to make ab initio gene predictions (22–25).

Methods to specifically predict CDSs in the transcriptome have also been developed and used for the characterization of large ESTs, cDNAs and RNA-seq collections (26–32). Similar to approaches used with genomic DNA sequences, CDSs annotation in high-throughput transcriptomic data include statistical and similarities metrics.

Typically, all computational calculations described above identify a single functional ORF or CDS per locus or transcript with a statistically significant signature of a protein-coding region. In this review, annotated CDSs are termed reference CDSs (refCDSs). When no substantial similarity is generated, the longest ORF is predicted to be the single most probable CDS and becomes the refCDS. Generally, a cutoff of 100 codons is applied for a significant CDS despite increasing evidence that peptides translated from short non-annotated CDSs have important functions (33–35). These rules, which exclude ‘small’ CDSs are part of the annotation guidelines used by virtually all publicly available gene sets, including AceView (36), GENCODE (37), RefSeq (38), Ensembl (39), VEGA (40), CCDS (41) and have exacerbated the single CDS dogma (Figure 1).

Establishing a comprehensive catalog of protein-coding genes and CDSs in the genome of eukaryotes remains a fundamental objective in modern biology and medicine, and the objective of identifying one complete refCDS for each gene is already an ambitious project (15). By reducing the search space in genome-wide analyses, the single refCDS principle greatly simplifies the annotation of CDSs and protein-coding genes but ignores additional CDSs and substantially underestimates the coding potential of genomes.

## A SECOND LOOK AT ORFs WITHIN EUKARYOTIC mRNAS

### Alternative ORFs versus reference CDSs

There are three reading frames for a transcript, and a typical mature mRNA may contain several alternative ORFs (altORFs) in addition to the refCDS (Figure 1). In this article, altORFs are defined as potential protein-coding sequences that are completely different from refCDSs in the same transcript. Proteins translated from altORFs have a different amino acid sequence and are not isoforms of the annotated proteins. AltORFs may be divided into three classes according to the location of the alternative versus the annotated TIS (Figure 1). Here, refCDSs are present in the +1 reading frame. AltORFs^5′UTR^ include altORFs with a TIS in the 5′ UTRs in any of the 3 reading frames. AltORFs that extend into refCDSs in the +2 or +3 reading frames are also considered to be AltORFs^5′UTR^. ORFs initiating upstream of the canonical initiation codon in the +1 reading frame code for long isoforms of the annotated proteins and will not be considered as altORFs in this article. AltORFs^5′UTR^ are also termed upstream ORFs. They repress the translation of refCDSs and are believed to be mainly translational regulatory elements (42–44). AltORFs^CDS^ include altORFs with a TIS inside the refCDSs in the +2 or +3 reading frame. AltORFs^CDS^ are also termed overlapping ORFs (45). AltORFs^3′UTR^ are localized inside 3′ UTRs.

As mentioned above, refCDSs are usually the longest CDSs (>100 codons), and altORFs are generally shorter. There are however exceptions when the first discovered protein translated from a specific mRNA does not turn out to be the largest one, such as the RPP14 mRNA. RPP14 refCDS is translated into a 124 amino acid subunit of the essential human ribonuclease P, and an altORF^3′UTR^ is translated into a 168 amino acid 3-hydroxyacyl-thioester dehydratase HsHTD2 which is important in mitochondrial fatty acid synthesis (46). The presence of both ORFs in the human RPP14 mRNA is clear evidence of a functional bicistronic human mRNA.

### ORFs within long non-coding RNAs

There is growing evidence that some annotated long non-coding RNAs (lncRNAs) are translated (47–52) and should in principle be classified as mRNAs. The homo sapiens apelin receptor early endogenous ligand lncRNA is a striking example of a previous lncRNA (RefSeq record, NR_038825.1) that recently changed status to mRNA (NM_001297550.1). This transcript codes for a conserved 32 amino acid hormone with critical function for cell movement and cardiovascular development (35,53). Similar to mRNAs, lncRNAs may be an important source of altORFs, but this review focuses on currently annotated protein-coding transcripts or mRNAs.

### *In silico* detection of altORFs

The serendipitous discovery that a few human genes express proteins from two different CDSs within a single mRNA prompted several laboratories to predict candidate altORFs (45,54,55). Three initial bioinformatics genome-wide studies predicted altORFs^CDS^ in mammalian transcripts (56–58). These studies used sequential filters with different stringencies, including a cut off size (150 or 500 nucleotides), conservation between species and the presence of a strong Kozak signal around the predicted TIS. Yet, they failed to predict several experimentally validated altORFs and subsequent research used less stringent filters and predicted 17,096 altORFs^CDS^ in human transcripts (59). These predictions were later applied to all classes of altORFs and in other eukaryotes (60). The human transcriptome contains 83,886 potential altORFs with a minimum size of 40 codons while the current human proteome contains about 52,000 annotated proteins (RefSeq release 72). A recently developed database specifically facilitates the detection of conserved altORFs^5′UTR^ completely located within 5′ UTRs (61). Overall, these analyses clearly detected potential protein-coding altORFs and revealed their widespread presence in different transcriptomes. These predictions likely underestimate the number of altORFs since the presence of an AUG initiation codon is an important criteria in the computational detection of altORFs. Yet, experimental evidence and evidence from evolutionary studies convincingly demonstrate that translation initiation does not always start at AUG codons (48,62–64).

## EXPERIMENTAL EVIDENCE FOR THE TRANSLATION OF altORFs

### Non-large scale approaches

Over the past two decades, several refCDS/altORF doublets have been discovered in mammals. These include INK4a/ARF (55), histone H4/OGP (65), XLalphas/ALEX (45,66), RPP14/HsHTD2 (46), PrP/altPrP (67), ATXN1/altATXN1 (68), A_2A_R/uORF5 (69), MKKS/uMKKS1 and uMKKS2 (70) and AT_1a_R/PEP7 (71). Remarkably and perhaps not coincidentatly, some alternative proteins functionally interact with their respective reference proteins. XLalphas/ALEX co-localize in plasma membrane ruffles and directly interact, and ALEX negatively regulates the activity of the G-protein XLalphas subunit (45,72). ATXN1/altATXN1 co-localize in nuclear foci and directly interact (68). The stimulation of A_2A_R by adenosine increases the expression of uORF5 through the activation of protein kinase A (69). PEP7 blocks the beta-arrestin dependent signaling pathway of AT_1a_R (71). We suggest that this may be a frequent or even common occurrence. This would provide a mechanism for ensuring that two or more proteins are always expressed together.

The co-expression of refCDS/altORF^CDS^ doublets in cultured cells transfected with an expression vector containing the refCDS is a significant result (67,68). First, it demonstrates that mechanisms for the translation of overlapping ORFs are constitutive and are an intrinsic feature of mammalian cells. The serendipitous discovery of the translation of HsHTD2 altORF^3′UTR^ in yeast cells transformed with an expression plasmid also indicates that translation mechanisms for altORFs are conserved in eukaryotes (46). Second, transfection of refCDSs in cultured cells for functional investigations of reference proteins is a common technique in research laboratories. The undetected co-expression of refCDS/altORF^CDS^ doublets indicate that the interpretation of some experimental results may have to be questioned (section Implications below).

Some human cancer specific antigens that are silent in normal tissues are also translated from altORFs^CDS^ (73–78). Such tumor-specific antigens are promising targets for the development of immunotherapies but the function of these proteins in cancer has not been characterized yet.

### Large scale approaches

#### Detection of alternative proteins by mass spectrometry

**Figure 2.**
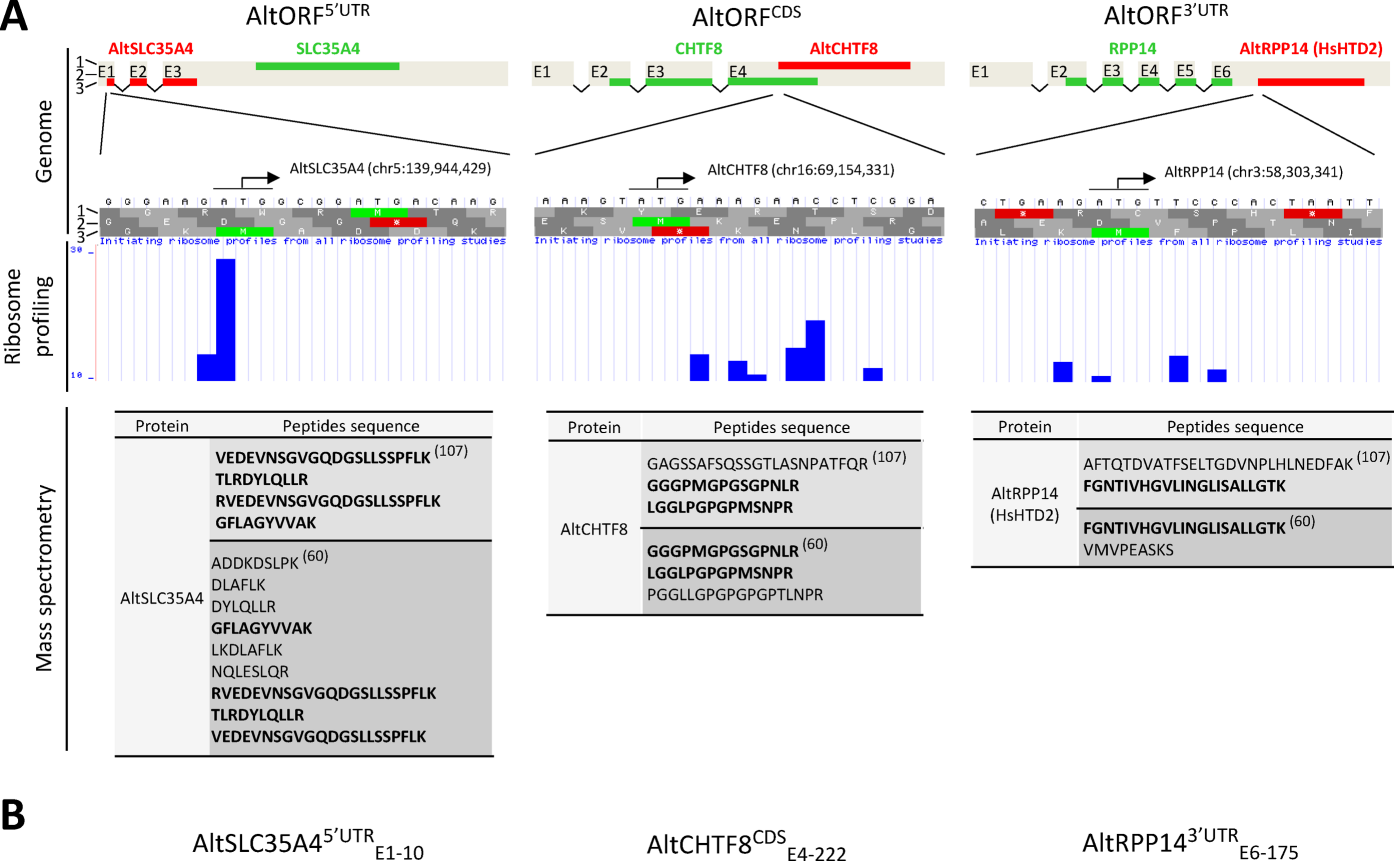
Ribosome profiling and MS are common techniques to detect alternative proteins in large scale studies. (**A**) AltSLC35A4, altCHTF8 and altRPP14(HTD2) are examples of alternative proteins detected by independent laboratories and at least by two different techniques. A schematic view of the genomic structures for the three human genes is shown with the chromosomal coordinates for the three alternative TISs. Exons (gray), refCDSs (green), altORFs (red), and the three reading frames are indicated. The genomic bases around the alternative TISs and the amino acids translated in the three reading frames are also indicated (image from the online genome browser GWIPS-viz (93)). Ribosome profiling data retrieved from GWIPS-viz indicate the position of initiating ribosome profiles from 4 profiling studies (62,63,90,136). Initiating ribosomes are clearly detected around altORFs TISs. The MS data show the peptide sequence of peptides detected in two independent studies, with peptides detected in both studies shown in bold (60,107). (**B**) Proposed nomenclature for altORFs. For example, altSLC35A4^5′UTR^_E1–10_ indicates an altORF in the SLC35A4 gene; it is located in the 5′ UTR, and the TIS starts at the 10th base of exon 1.

Two main challenges have hampered the detection of alternative proteins by mass spectrometry (MS). First, protein sequence databases are central to the success of MS-based protein identification. UniProt Knowledgebase (79) and the NCBI Reference Sequence collection (38) are among the most widely used databases. Alternative proteins are not annotated in these databases and, as such, cannot be detected and remain invisible to MS users. We addressed this issue by creating a database containing the protein sequence of all predicted altORFs. A total of 1259 novel alternative proteins were detected in different human cell lines, tissues and fluids (60) (Table 1 and Figure 2A).

**Table 1.** Examples of detected alternative proteins in the literature

altORF^5′UTR^	altORF^CDS^	altORF^3′UTR^
AltSMCR7L (60,62,107,108)	AltCHTF8 (60,62,107)	HsHTD2 (46,60,89,107)
AltSLC35A4 (60,62,107,108)	AltHNRPUL1 (60,107)	AltSF1 (60,107)
uORF5 (69)	AltATXN1 (68)	
PEP7 (71)	AltPrP (67)	
uMKKS1 and 2 (70)		

Second, altORFs are shorter than refCDSs and small proteins in general are more challenging to detect by MS. In an approach specifically designed for the detection of non-annotated small proteins, a total of more than 200 polypeptides of less than 150 amino acids long were detected in three independent studies performed in human K562 cells and tissues (80–82). The coding sequences were searched against public or in-house built cDNA libraries and matched to all three classes of altORFs.

#### Detection of altORFs being translated by ribosome profiling

Ribosome profiling is an emerging technique that provides genome-wide information on different aspects of translation in vivo (83–85). Briefly, ribosome-bound mRNAs are isolated and treated with a nuclease. The resulting ribosome-protected RNA fragments or footprints are identified by high throughput sequencing. This novel technology can map TISs and regions within transcripts that are translated, and has already provided major novel insights into the complexity of translated sequences and the mechanisms of protein synthesis and translational control (84,86). Importantly, ribosome profiling detects functional TISs independently of annotated CDSs and is thus completely unbiased towards the detection of annotated CDSs or alternative CDSs.

In mouse embryonic fibroblast cells and in human HEK293 cells, the majority of mRNAs contain more than one TIS and >50% of detected TISs map to altORFs^5′UTR^ and altORFs^CDS^. A small proportion of TIS are detected in 3′ UTRs (48,62). *Ab initio* predictions of transcriptome-wide TISs using a neural network trained on ribosomal profiling data generated in a human monocytic cell line independently confirmed these results (63). In violation of the single CDS dogma, these initial striking observations on the widespread translation of altORFs attest to the presence of an unanticipated mechanism for protein diversity. Extensive translation of altORFs have been reported in different organisms and under various experimental settings (47,51,87–92) (Table 1 and Figure 2A). Functional annotated and alternative TISs detected by ribosomal profiling are now mapped and easily searchable in several databases and online genome browsers (93–96).

Few altORFs^3′UTR^ were detected in the studies cited above, but this is not surprising given that the ribosome profiling technology used was very inefficient at detecting ribosomes in the 3′ UTRs (97). Recently, ribosome profiling detected 3′ UTR translation in yeast cells, but TISs were not investigated (98).

## TRANSLATION MECHANISMS FOR ALTERNATIVE PROTEINS

AltORFs are clearly not receiving sufficient attention in genome annotations, and the translation of altORFs does not comply with the single CDS rule. Yet, a large number of altORFs are translated and cellular translational mechanisms must operate which allow more than one protein to be translated from a single mRNA species.

The scanning mechanism for initiation of translation predicts that altORFs^5′UTR^ TISs, strategically located in 5′ UTRs are detected prior to the annotated TISs, particularly if they have a strong Kozak sequence (2,4,99). For such mRNAs, altORFs^5′UTR^ (also termed upstream ORFs) are translated first. The translation of refCDSs relies on a reinitiation mechanism highly dependent on the length of altORFs^5′UTR^, the distance and structural constraints between altORFs^5′UTR^ and the refCDSs (43,100–105). Two factors essential for translation reinitiation of refCDSs after translation of altORFs^5′UTR^ with a strong Kozak sequence were recently identified in drosophila cells (106). Based on results obtained with the human immunodeficiency virus type 1 tat mRNA, it was predicted that altORFs^5′UTR^ longer than 55 codons would prevent reinitiation (105).

Nevertheless, SLC35A4 contains 11 ORFs upstream of the refCDS, and translation of the 102 codon long 11th upstream ORF was independently detected by several groups by ribosomal profiling and MS (60,107,108). Clearly, other mechanisms may operate for the translation of such altORFs and for altORFs^CDS^ and altORFs^3′UTR^ for which several upstream TISs must be bypassed before ribosomes reach the altORF TIS. One such possible mechanism is leaky scanning. Leaky scanning allows for ribosomes to scan through TISs without initiating translation and results in the translation of downstream ORFs (100). An optimal Kozak context strongly reduces but does not completely prevent leaky scanning in all mRNAs. A large scale analysis of 22 208 human mRNAs indicates that only 37.4% of mRNAs have an annotated TIS with an optimal Kozak sequence. The majority of human mRNAs are thus predicted to undergo leaky scanning of annotated TISs and produce alternative proteins (109). The osteogenic growth peptide represents a fascinating example of a biological active molecule translated from an altORF^CDS^ by leaky scanning (65,109). The corresponding refCDS encodes the small 103 amino acids and extremely well conserved histone H4, yet it shelters an altORF. Several CDSs in polycistronic viral RNAs also use a leaky scanning mechanism for their translation in mammalian cells (110). Overall, leaky scanning is a well-recognized translational mechanism which allows ribosomes to reach downstream altORFs^CDS^ and altORFs^3′UTR^ TISs in cellular mRNAs.

In addition to leaky scanning, viruses have evolved other strategies to make eukaryotic cells translate several CDSs in polycistronic mRNAs using non-canonical mechanisms (110–113). Internal ribosomal entry sites (IRES) are structured RNA sequences able to recruit the translation machinery under conditions in which cap-dependent translation is compromised. Although the function of IRES in animal and plant viral RNAs is well established, the presence of putative IRES in cellular mRNAs is disputed in the literature and it would be too premature to invoke this mechanism for the translation of altORFs (114–116). Ribosome shunting involves conventional cap-dependent initiation, but the scanning ribosome bypasses a large structured region of the mRNA to reach downstream TISs (110). Two human mRNAs use ribosome shunting for the translation of their refCDSs, HSP70 and cIAP2 (117,118). Ribosome shunting would provide a possible mechanism allowing scanning ribosomes to reach altORFs^CDS^ and altORFs^3′UTR^. Ribosome shunting depends on ribosomal protein S25 (119). Stable ribosomal protein S25 knockdown cells are viable and thus it should be possible to verify the function of that protein in the translation of altORFs.

3′ UTRs are a large source of potential altORFs and a majority of alternative proteins detected by MS are encoded in 3′ UTRs (60). A recent ribosome profiling investigation revealed a high abundance of ribosomes in 3′ UTRs of drosophila and human cells but this study did not provide evidence of actual 3′ UTR translation (97). In yeast cells, ribosome profiling and MS demonstrated 3′ UTR translation through an Rli1-dependent mechanism (98).

Does the translation of altORFs necessarily violate the cap-dependent scanning model? This model for the selection of TISs in eukaryotic mRNAs has provided a fundamental framework for the study of the regulation of translation initiation in eukaryotes, and is supported by a substantial body of data (2–4). A stringent scanning mechanism states that 5′ proximal ORFs are preferentially translated and downstream ORFs are thus unlikely to be translated. More than 60% of human mRNAs do not have an annotated TIS with an optimal Kozak sequence (109), and half of human mRNAs have at least one altORF^5′UTR^ (43). Thus, a strict scanning mechanism could not sustain the translation of refCDSs in a large portion of human mRNAs. For that reason, leaky scanning and reinitiation mechanisms have been incorporated into the scanning model (5,100,120). In addition, reinitiation mechanisms are particularly efficient in mammalian cells. The mechanism of translation for bicistronic GDF1/CERS1, SNRPN/SNURF and RPP14/HsHTD2 was not investigated but is likely to occur by leaky scanning and/or reinitation (46,121,122). Functional polycistronic mRNAs were discovered in metazoans (123–126). For one of them, translation clearly occurs via leaky scanning (123). Overall, there is strong evidence that translation of altORFs in eukaryotic mRNAs does not violate but requires some already demonstrated plasticity of the scanning rule.

In addition to the widely accepted scanning model, mechanisms such as ribosomal tethering, clustering, internal initiation and RNA looping have been proposed for the recruitment of ribosomes to TIS (127–129). For example, two structural elements in the histone H4 mRNA refCDS tether the cap and the 43S preinitiation complex, respectively, and detection of the reference TIS is scanning-independent (128). Importantly, these scanning-independent mechanisms bypass 5′ TISs and allow the recognition of downstream TISs, and possibly the translation of altORFs^CDS^ and altORFs^3′UTR^. These studies were mostly performed with model mRNAs and provided proof of principle that scanning-independent mechanisms can operate. Yet, it will be important to determine if these mechanisms are involved in the translation of cellular mRNAs.

## TRANSLATION OF ALTERNATIVE PROTEINS: IMPLICATIONS

The observation that expression of AltORFs may well be a widespread phenomenon has vast implications regarding the way the structure and function of genes are investigated, and the way ‘omics’ data are interpreted. In mammalian mRNAs, most altORFs with a minimum size of 40 codons are distributed either in 3′ UTRs (46%) or inside refORFs (41%) (60). The presence of a large number of altORFs nested inside refCDSs has the potential to generate results which are difficult to interpret since plasmid driven refCDSs expression in cultured cells or transgenic animals may result in the unnoticed co-expression of altORFs (60,67,68,130). Conversely, knocking down mRNAs could inhibit the expression of both annotated and alternative proteins (60,67,68). The human coagulation factor IX contains an altORF^CDS^ encoding a 64 amino acid protein. Unfortunately, the co-expression of this alternative protein from a transgenic therapeutic cassette elicits an unwanted cytotoxic T lymphocyte response, resulting in the death of cells expressing the transgene (130). The authors suggested that alternative TISs should be inactivated in transgenes used for therapeutic purposes.

Large scale screening assays, including yeast 2-hybrid assays, result in the identification of out-of-frame clones that are systematically rejected. Yet, some may represent positive hits such as the interaction between BRCA1 and altMRVI1 (60).

Other potential implications particularly for overlapping refCDSs/altORFs are more speculative but surely diserve further investigations. Functional polymorphisms affecting the overlapping XLalphas/ALEX refCDS/altORF^CDS^ doublet associate with G signaling hyperfunction in the platelets of patients (72). Deregulated signaling results in part from a decreased interaction between the G-protein XLalphas subunit and ALEX. In addition, synonymous nucleotide substitutions in coding regions or silent mutations are implicated in a number of pathological manifestations (131). A fraction of synonymous mutations in the refCDS may produce a nonsynonymous mutation in an overlapping altORF^CDS^ and alter the corresponding alternative protein.

## CONCLUSION AND FUTURE PERSPECTIVES

Eukaryotic mRNAs contain a single refCDS but usually contain several ORFs. The combination of the traditional view of a monocistronic mRNA with computational approaches for genome-wide annotations rejected altORFs. Unfortunately, the single CDS dogma has artificially limited our view of the coding capacity of mRNAs and has prevented the discovery of alternative proteins despite some clues in the literature over the years (132). Recently, a large and rapidly growing body of evidence has provided conclusive experimental evidence for the translation of alternative proteins in addition to the annotated protein from the same mRNA (133).

A new field of investigation is emerging and will have to address several issues. The contribution of alternative proteins to the translational landscape will need to be determined and will likely take advantage of the combination of ribosome profiling and MS-based proteomics (134). In order to demonstrate the functional relevance of alternative proteins, it will be important to specifically inactivate altORFs without affecting refCDSs using gene editing techniques now available (135). New tools such as specific antibodies, updated databases to keep track of the discovery of new transcripts, and new methods to study small proteins will have to be generated. Functional relationships between reference and alternative proteins expressed from the same gene may help identify a new layer of regulation of protein activity (45,68,72).

In parallel to the growing evidence for the translation of non-annotated CDSs, the coding potential of genomes should be updated and the nomenclature for alternative ORFs should be standardized. These unconventional ORFs are termed altORFs (60), overlapping ORFs (56), uORFs (42), sORFs (33), and smORFs (91). The nomenclature for the corresponding translational products is also confusing, and there is a clear need to adopt a standard classification. sORFs and smORFs are shorter than 100 codons and do not represent all non-annotated ORFs. Similarly, uORFs (altORFs^5′ UTR^) and overlapping ORFs (altORFs^CDS^) do not include ORFs located in 3′ UTRs. We suggest the term alternative ORFs or altORFs to distinguish all the currently non-annotated ORFs from the annotated CDSs (Figure 1). As indicated above, altORFs may be divided into three classes, altORF^5′UTR^, altORF^CDS^, and altORF^3′UTR^ according to the localization of the TIS in the tripartite structure of the longest mRNA. Since several altORFs may be present within one mRNA, we propose to add the exon containing the alternative TIS and the position of the first base of the TIS relative to the first base of this exon (Figure 2B). AltORFs in non-coding RNAs may be termed altORFs^nc^.
